# LDL transcytosis passes through the *trans*-Golgi network and requires Rab10

**DOI:** 10.1016/j.jlr.2025.100893

**Published:** 2025-09-02

**Authors:** Tse Wing Winnie Ho, Changsen Wang, Warren L. Lee

**Affiliations:** 1 Keenan Centre for Biomedical Research, St. Michael’s Hospital, Toronto, Canada; 2 Department of Laboratory Medicine and Pathobiology, University of Toronto, Toronto, Canada; 3 Department of Biochemistry, University of Toronto, Toronto, Canada; 4 Department of Medicine and the Interdepartmental Division of Critical Care Medicine, University of Toronto, Toronto, Canada

**Keywords:** LDL, Golgi apparatus, vascular biology, lipoproteins, atherosclerosis, transcytosis, Rab10, Rab6

## Abstract

Atherosclerosis begins with the subendothelial retention of LDLs from the circulation. While LDL transcytosis across the endothelium is mediated by scavenger receptor class B type I and activin-like kinase receptor 1 and is usually independent of LDL receptor, the intracellular mechanisms and route of LDL transcytosis remain unclear. Using total internal reflection fluorescence microscopy in LDL receptor-depleted human coronary artery endothelial cells, we found that LDL transcytosis can proceed both directly and indirectly from an intracellular compartment. During LDL transcytosis, LDL was observed to colocalize with the Golgi apparatus over time, specifically with the *trans*-Golgi network marker TGN46. Systematic examination of endothelial Rab proteins known to regulate Golgi traffic identified Rabs 6a and 10 to be required for LDL transcytosis. Depletion of Rab10 or Rab6a significantly inhibited LDL transcytosis but had no effect on albumin transcytosis. Expression and localization of scavenger receptor class B type I and activin-like kinase receptor 1 were also unimpaired. Conversely, overexpression of Rab10 increased LDL transcytosis. Finally, depletion of Rab10 increased colocalization of LDL with the *trans*-Golgi network and led to expansion of the Golgi, indicative of impaired exocytosis from the Golgi. However, colocalization of Rab10 with LDL did not increase over time, and Rab10 did not accumulate at the base of the cell, suggesting its role is specifically related to LDL exit from the Golgi rather than direct transport. In summary, during LDL transcytosis, internalized LDL is transported to the Golgi, which serves as a reservoir of LDL that can undergo exocytosis. Our results identify specific Rab proteins as critical regulators of this process.

Although atherosclerosis begins with the retention of lipoproteins in the subendothelial intima of arteries, how circulating lipoproteins cross the endothelium to enter the artery wall has long been controversial. The average diameter of LDL particles is too large to pass through competent endothelial cell-cell junctions, and the endothelium overlying early atheromatous lesions appears both healthy and intact (1). Advances in live cell imaging have since confirmed that LDL can traverse individual endothelial cells by transcytosis, a vesicular transport process that starts with binding of LDL at the apical cell surface by the scavenger receptor class B type I (SR-BI) (2) and the activin-like kinase receptor 1 (ALK1) (3). Both receptors are enriched in caveolae, membrane microdomains characterized by cholesterol, sphingolipids, and the protein caveolin-1. Conversely, the high-affinity LDL receptor (LDLR) is not normally required. Mice with endothelial-specific deletion of SR-BI (4) and ALK1 demonstrate reduced transcytosis of LDL and are protected from atherosclerosis. Furthermore, the inhibition of ALK1 using a novel antibody that prevents ALK1-LDL binding has been demonstrated to both prevent incipient disease and to accelerate regression of established arterial plaques. Thus, LDL transcytosis is now recognized as a bona fide therapeutic target (5).

Despite these recent advances, very little is known about the mechanisms of LDL transcytosis beyond the cell surface. While the fate of LDL after its internalization by LDLR has been extensively characterized, the fact that LDLR is not required for transcytosis suggests that a distinct intracellular route may be involved. Elucidating the intracellular route taken by LDL during transcytosis may identify potential therapeutic targets for the prevention of atherosclerosis.

Here, we demonstrate that LDL transcytosis by coronary artery endothelial cells continues for at least an hour after its internalization, indicating its retention in an intracellular niche. Transcytosis is independent of the canonical endosome maturation pathway, as depletion of Rabs5a and 7 had no impact. Instead, after its internalization, LDL localizes to the *trans*-Golgi network. The exit of LDL from this unsuspected intracellular niche requires the GTPase Rab10 in order for transcytosis to continue.

## Materials and methods

### Cell culture

Primary human coronary artery endothelial cells (HCAECs) were purchased from PromoCell (Heidelberg, Germany). Human microvascular endothelial cells (HMEC-1) were obtained from the Centers for Disease Control and Prevention. Cells were cultured and maintained in Lonza Endothelial Cell Basal Medium-2 (CC-3156; Lonza) and EGM-2 SingleQuots Supplements (CC-4147) without gentamicin in an incubator at 5% CO_2_ and 37°C. Media were changed every 3–4 days. In all experiments, cells were used in passages 5–9.

### LDL isolation and labeling

Blood was obtained from healthy donors with informed consent and in accordance with a protocol approved by the Unity Health Toronto Research Ethics Board (REB 19-314). The isolation and labeling of LDL with Alexa Fluor 568 NHS Ester Succinimidyl Ester (A20003; ThermoFisher), Alexa Fluor 488 NHS Ester Succinimidyl Ester (A20000; ThermoFisher), or DiI (ab145311; Abcam) were conducted as previously described (6).

### Transfection

HCAECs were transfected at 80–90% confluency using jetOPTIMUS Transfection Reagent (101000025 and 201000001; Polyplus). A master mix of 200 μl jetOPTIMUS buffer, 2 μl jetOPTIMUS reagent, and 2 μg of Rab10-EGFP plasmid (49472; AddGene) was made according to the manufacturer’s instruction and added dropwise to HCAECs on coverslips in fresh complete serum EGM-2. After 4 h, fresh media were replaced, and experiments were performed 24 h after transfection when cells were confluent. After the experiment, cell lysates were collected to confirm overexpression.

### Knockdown

Knockdown was performed on HCAECs at 70% confluency 48 h prior to the experiment. A master mix with 300 μl Opti-MEM (31985070; ThermoFisher), together with 4 μl Lipofectamine RNAiMAX (13778075; ThermoFisher), and 20 nM of negative (1027310; Qiagen), SR-BI (GS949, 1027417; Qiagen), or LDLR (GS3949: SI05587484, SI03024525, SI00011186, and SI00011179), ALK1 (GS94: SI02758392, SI02659972, SI04947999, and SI04894687), Rab4a (GS5867: SI02662786, SI02662226, SI02655030, and SI00301581), Rab5a (GS5868: SI02655037, SI00301588, SI03111115, and SI03046673), Rab6a (GS6870: SI02655044, SI02654120, SI02654036, and SI04437006), Rab7a (GS7879: SI02662240, SI03104255, SI03096016, and SI00066409), Rab8b (GS51762: SI02662814, SI02662261, SI03023972, and SI00116501), Rab10 (GS10890: SI00301546, SI03037076, SI00113715, and SI00113708), Rab11a (GS8766: SI02663206, SI02655247, SI00301553, and SI04437881), Rab18 (GS22931: SI02662709, SI02662156, SI03045161, and SI00130494) FlexiTube GeneSolution siRNAs (1027416; Qiagen) were prepared according to the manufacturer’s instructions and added to cells in fresh media. Fresh media were replaced 24 h after transfection. Experiments were performed 48 h after transfection. Cell lysates were collected after the experiment to confirm knockdown by Western blot.

### Total internal reflection fluorescence microscopy

To assess LDL transcytosis, total internal reflection fluorescence (TIRF) microscopy was performed as previously described (2, 6, 7). Briefly, HCAECs were first seeded onto glass coverslips, and experiments were performed when cells were confluent. For the brefeldin A (BFA) experiment, cells were treated with 20 μg/ml of BFA (11861; Cayman Chemical) in warm complete media for 1 h at 37°C. During the TIRF assay, cells were washed with PBS and incubated with 20 μg/ml of DiI-LDL, Alexa Fluor 488-conjugated LDL (Alexa 488-LDL), or Alexa Fluor 568-conjugated LDL in cold HPMI (Hepes-buffered RPMI, 22400-089; Gibco) for 10 min at 4°C, allowing apical binding of LDL to endothelial cells. To analyze albumin transcytosis, 20 μg/ml of Alexa Fluor 488-conjugated albumin was incubated instead for 10 min at 4°C. After 10 min, excess unbound ligand was washed off by rinsing with PBS 3 times, and cells were incubated with warm HPMI in a live cell imaging chamber at 37°C. To stain the nuclei of the confluent layer of cells, cells were incubated with 1:200 of NucBlue Live Ready Probes Reagent (R37605; ThermoFisher). Cells were then imaged using the Quorum Diskovery TIRF Unit with Hamamatsu ImagEM X2 EM-CCD camera, 63x oil objectives (1.47, CORR TIRF): 405 nm, 488 nm, 561 nm, and 637 nm laser lines and 450/50, 525/50, 600/50, 610/75, and 700/75 emission filters. The basal membrane of individual cells was imaged by TIRF, and image acquisitions were acquired using Quorum acquisition software (Quorum, Canada). At least 10 or more randomly selected cells per condition were imaged in each experiment. To quantify transcytosis events in real time, videos consisting of 150 frames were captured with 100 ms exposure per frame. Transcytosis events were defined as vesicle exocytosis events at the basal membrane, marked by a rapid decrease of fluorescence intensity upon vesicle fusion with the basolateral membrane, as previously described (8). These events were analyzed using a single-particle MATLAB tracking algorithm.

For the long-term pulse-chase experiment, cells were incubated with LDL, as mentioned above. After removing the excess unbound ligand, cells were chased in warm HPMI for 0 min, 10 min, 20 min, 30 min, 40 min, 50 min, 60 min, and 70 min at 37 °C before imaging. Before each of the time points, fresh media were replaced to prevent potential recycling of exocytosed LDL during the experiment. In each experiment, at least five or more randomly selected cells per condition were imaged.

For the dual pulse-chase experiment, in which static analysis was required, cells were first incubated with 20 μg/ml of DiI-LDL at 4°C for 10 min. After washing, cells were chased in warm HPMI in an incubator at 37°C for 30 min. Cells were then washed and incubated with 20 μg/ml of Alexa 488-LDL at 4°C for 10 min before washing and imaging. Still images were acquired at the TIRF zone (110 nm from the bottom of the cell) to analyze colocalization between LDL vesicles pulsed at 2 different time points in each cell and to quantify Rab10 intensity near the basal membrane of the cell.

### Uptake assay

To analyze LDL internalization, HCAECs were serum starved in a 37°C incubator prior to the experiment. After 30 min, cells were incubated with 150 μg/ml of Alexa568-LDL in warm HPMI for 10 min at 37°C. For the dual pulse-chase whole cell experiment, cells were incubated with DiI-LDL and Alexa 488-LDL as described above. For dextran uptake, cells were incubated with 100 μg/ml of dextran (D1818; Invitrogen) for 15 min at 37°C. For the LysoTracker experiment, cells were incubated with 25 nM of LysoTracker before imaging. Cells were then washed with PBS 3 times, followed by fixation with 4% paraformaldehyde (PFA, 15710; Electron Microscopy Sciences) for 15–20 min at room temperature. After washing, cells were incubated with 100 mM glycine for 20 min. The coverslips were mounted on slides with Dako Fluorescence Mounting Medium (S3023; Agilent) containing 1:5,000 4',6-diamidino-2-phenylindole (DAPI) (10236276001; Roche). Negative controls without LDL incubation were included in all experiments.

LDL uptake was imaged using spinning-disk confocal microscopy with Quorum Diskovery/Nipkow Spinning Disc by ORCA-Flash 4.0 V2 PLUS sCMOS camera, 63x/1.47 (oil) objective: 405 nm, 488 nm, 561 nm, and 637 nm laser lines. At least 10 randomly selected fields of cells were imaged and acquired with a z-stack interval of 0.25 μm for each condition in each experiment. In each experiment, all microscope settings were kept constant between conditions. The background fluorescence intensity from the blank was subtracted prior to analysis.

### Immunofluorescence

HCAECs were washed and fixed with 4% PFA at room temperature for 15 min. After washing 3 times with PBS, PFA was neutralized with 100 mM glycine in PBS for 20 min, followed by permeabilization with 1% BSA and 0.1% Triton X-100 (TRX506.500; Bioshop) in PBS for 20 min. Cells were then blocked with 5% BSA for 20 min at room temperature after washing. For cell surface immunofluorescence experiments, cells were blocked after neutralization, and no permeabilization was performed. Immunostaining was performed with 1:50 mouse anti-SR-BI (sc-518140; Santa Cruz Biotechnology), goat anti-ALK1 (AF370; R&D Systems), rabbit anti-Rab10 (8127; Cell Signaling), mouse anti-TGN46 (66477-1-Ig; Proteintech), rabbit anti-GM130 (NBP2-53420; Novus), rabbit anti-ApoB (NBP2-38608; Novus), rabbit anti-Claudin 5 (ab15106; Abcam), 1:25 mouse anti-early endosome antigen 1 (sc-137130, AF647; Santa Cruz Biotechnology), mouse anti-CD71 (sc-65882, AF488; Santa Cruz Biotechnology), mouse anti-LDLR (sc-18823, AF647; Santa Cruz Biotechnology), and/or 1:15 mouse anti-Golgi (sc-58770, AF488; Santa Cruz Biotechnology) primary antibodies in 1% BSA for 1 h at room temperature. After washing, the coverslips were incubated with 1:1,000 of Alexa Fluor 488 anti-rabbit antibody (111-545-003; Jackson ImmunoResearch), Alexa Fluor 488 anti-mouse antibody (115-546-006; Jackson ImmunoResearch), Alexa Fluor 555 anti-goat antibody (A32816; Invitrogen), and/or Alexa Fluor 647 anti-rabbit secondary antibodies (111-605-003; Jackson ImmunoResearch) for 1 h at room temperature. After washing, the coverslips were mounted on slides with DAPI-containing (1:5,000) Dako Fluorescence Mounting Medium. For LDL and Golgi colocalization experiments, the nucleus was stained with 1 μg/ml of DAPI prior to mounting with Dako Fluorescence Mounting Medium. Negative controls without primary antibody incubation were included in all experiments where applicable.

Z-stack images were acquired by spinning-disk confocal microscopy by Quorum Diskovery/Nipkow Spinning Disc, ORCA-Flash 4.0 V2 PLUS sCMOS camera with 63x/1.47 (oil) objective: 405 nm, 488 nm, 561 nm, and 637 nm laser lines. For each condition, 10 or more randomly selected fields of cells with a z-stack interval of 0.25 mm were imaged in each experiment. Microscope settings were kept constant within each experiment. After image acquisition, background fluorescent intensity was excluded using Volocity 6.3 software (Quorum). For image analysis, fluorescence intensity was quantified using ImageJ software (Fiji) and normalized to the number of nuclei in each field.

To analyze colocalization, threshold settings were kept constant between conditions in each experiment, and colocalization was quantified using Pearson’s coefficient (for dual LDL pulse-chase experiments) or Mander’s coefficient on Volocity software. Pearson’s coefficient was used when correlation between 2 signals was of interest; Mander’s coefficient was used when the specific fraction of the total intensity of a fluorophore that overlaps with another fluorophore was of interest.

To perform 3D reconstruction of the images, 3D rendering and visualization of the images were performed on Imaris 9.6.0 software (Oxford Instruments). All parameters for 3D modeling were kept constant to allow comparisons between experimental conditions.

### Western blot

Cell lysates were collected with SDS-containing lysis buffer (62.5 mM Tris-HCl [pH 6.8%], 2% SDS, 10% glycerol, and 10 mM DTT). Lysates were resolved on a polyacrylamide gel before transferring to a nitrocellulose membrane. Blots were then blocked using 5% BSA in TBS with 0.1% of Tween-20 (TBST) for 1 h at room temperature. After washing, blots were incubated with primary antibodies diluted in 1:1,000 overnight at 4°C. Immunoblotting was performed with the following primary antibodies: goat anti-LDLR (AF2148; R&D Systems), rabbit anti-SR-BI (NB400-104; Novus Bio), rabbit anti-ALK1 (70R-49334; Fitzgerald), mouse anti-β-actin (sc-47778; Santa Cruz Biotechnology), rabbit anti-Rab1a (PA5-44578; Invitrogen), rabbit anti-Rab4a (2167; Cell Signaling), rabbit anti-Rab5a (ab218624; Abcam), rabbit anti-Rab6a (10187-2-AP; Proteintech), mouse anti-Rab7 (sc-376362; Santa Cruz Biotechnology), rabbit anti-Rab8a (6975; Cell Signaling), rabbit anti-Rab8b (11792-1-AP; Proteintech), rabbit anti-Rab10 (8127; Cell Signaling), mouse anti-Rab11a (sc-166523; Santa Cruz Biotechnology), rabbit anti-Rab18 (11304-1-AP; Proteintech), rabbit anti-Calnexin (2679; Cell Signaling), mouse anti-VE-cadherin (sc-9989; Santa Cruz), rabbit anti-ApoB (ab20737; Abcam), rabbit anti-GM130 (A5344; ABclonal), rabbit anti-TGN46 (A16707; ABclonal), and rabbit anti-DRP1 (12957-1-AP; Proteintech). After washing with TBST 3 times, blots were incubated with 1:10,000 of HRP-conjugated secondary antibodies (P0449 goat, DAKO; 7076 mouse, 7074 rabbit, Cell Signaling Technology) for 1 h at room temperature. After washing with TBST, blots were incubated with Clarity Western ECL Substrate (1705060; Bio-Rad) for 5 min at room temperature and visualized by chemiluminescence with the ChemiDoc Imaging System (Bio-Rad). Band intensity was quantified using Image Lab (Bio-Rad) and normalized to the loading control.

### Subcellular fractionation

Because of the very high numbers of cells required for subcellular fractionation, immortalized HMEC-1 cells were used. HMEC-1s were grown in 10-cm dishes to 70% confluency before knocking down LDLR for 48 h as described above. To analyze LDL subcellular localization, HMEC-1 cells were starved in serum-free media for 30 min and then incubated with 300–400 μg/ml of LDL in warm serum-free media for 1 h in a 37°C incubator. Cells were washed with PBS 3 times and chased in warm complete media for 20 min.

All the following steps were performed on ice. Before homogenization, cells were washed twice with cold PBS and 1X homogenization buffer (0.25 M sucrose, 1 mM EDTA, 10 mM and Hepes [pH 7.4]). Cells were then scraped off into homogenization buffer with 1X protease inhibitor cocktail (11836170001; Roche) and disrupted by 20 strokes in a Dounce homogenizer followed by five passages through a 27-gauge needle. The homogenate was centrifugated at 1,500 *g* for 10 min at 4°C. The postnuclear supernatant was collected and centrifuged using an SW55 Ti rotor at 35,000 rpm for 1 h at 4°C. The membrane pellet was resuspended in 0.8 ml of homogenization buffer with protease inhibitor. A discontinuous iodixanol gradient from 2.5% to 30% was made with iodixanol and homogenization buffer with protease inhibitor (800 μl 2.5%, 1.6 ml 5%, 1.6 ml 7.5%, 1.6 ml 10%, 400 μl 12.5%, 1.6 ml 15%, 400 μl 17.5%, 400 μl 20%, and 400 μl 30%). Gradients were then set up and loaded into 13 ml polypropylene ultracentrifuge tubes (331372; Beckman Coulter) and centrifuged in an SW41 Ti rotor at 40,000 rpm for 2.5 h at 4°C. The resulting gradient was collected in fractions of 800 μl. Proteins were extracted using cold acetone and SDS-PAGE, and Western blot was performed as described above.

### Statistics

All experiments were performed at least three times independently, unless otherwise indicated. Data were presented as mean with error bars representing the SEM. Statistical comparisons were performed using GraphPad Prism software, version 9 (GraphPad Software, Inc, La Jolla, CA). Comparisons between 2 groups were performed using a two-tailed Student's *t*-test or Mann-Whitney *U* test depending on if normality was established. For comparisons with 3 or more groups, one-way ANOVA was performed, followed by Bonferroni’s or Dunn’s multiple comparison test depending on the normality of the data. Statistical analyses between groups were considered as statistically significant with a *P* value of less than 0.05.

## Results

### Transcytosis of LDL persists for over an hour after an acute pulse of LDL

**Fig. 1:**
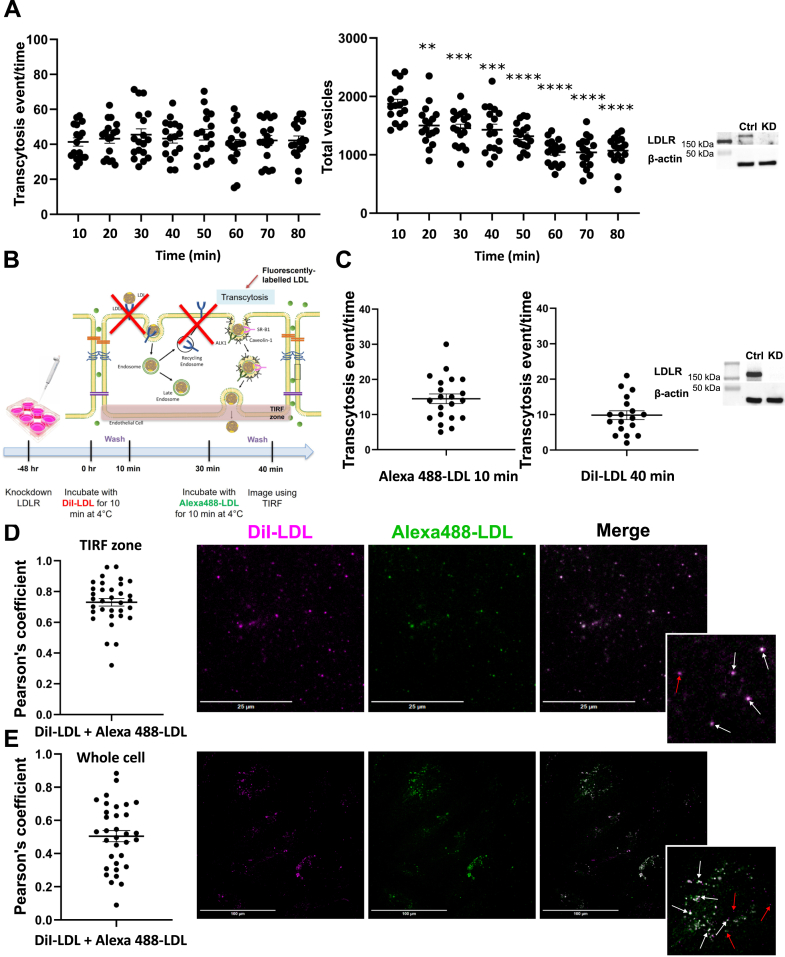
HCAECs are able to retain LDL intracellularly and undergo transcytosis over time. A: HCAECs depleted of LDLR exhibited ongoing Alexa Fluor 568-conjugated LDL transcytosis even as the number of Alexa-LDL-containing vesicles at the base of the cell decreases over time. Left shows quantification of transcytosis events, middle shows the number of vesicles in the TIRF zone, and right shows representative Western blot to confirm LDLR knockdown. Each point represents one cell. n = 3. ∗∗*P* < 0.01, ∗∗∗*P* < 0.001, ∗∗∗∗*P* < 0.0001. B: Timeline of the dual pulse-chase experiment. HCAECs were knocked down with LDLR 48 h before the experiment. Cells were first incubated with DiI-LDL for 10 min, followed by a 20 min chase. Subsequently, cells were incubated with Alexa 488-LDL for 10 min before imaging with TIRF microscopy. C: Transcytosis events of Alexa 488-LDL and DiI-LDL pulsed at different time points within the same cell after the depletion of LDLR. Left shows quantification of transcytosis events, right shows representative Western blot to confirm LDLR knockdown. Each point represents one cell. n = 3. D: Colocalization of DiI-LDL and Alexa 488-LDL vesicles pulsed at different time points in HCAECs depleted of LDLR, imaged at the base of cells within the TIRF zone using TIRF microscopy (Pearson’s coefficient = 0.730). Quantification of colocalization is on the left, and representative images are on the right. n = 3. The scale bar represents 25 μm. E: Colocalization of DiI-LDL and Alexa 488-LDL vesicles pulsed at different time points in HCAECs depleted of LDLR, imaged using confocal microscopy (Pearson’s coefficient = 0.505). Quantification of colocalization is on the left, and representative images and magnified insets are on the right. White arrows point toward areas with colocalization, red arrows point toward areas without colocalization. n = 3. The scale bar represents 100 μm. Statistical significance was assessed by one-way ANOVA followed by Dunn’s (transcytosis events) or Dunnett’s (total vesicles) multiple comparison test; error bars represent SEM.

To observe the kinetics of LDL transcytosis, we allowed a confluent monolayer of HCAECs to bind Alexa488-LDL at 4°C, preventing internalization (9). Unbound LDL was then removed by rinsing, followed by measurement of LDL transcytosis at 37°C by TIRF microscopy at intervals of 10 min. As LDLR is not required for basal transcytosis (2, 4), cells had previously been depleted of LDLR by siRNA to eliminate confounding from the LDLR-dependent endocytic pathway. The media of the cells were also changed at each time point to reduce confounding by recycling of exocytosed LDL. As previously reported, transcytosis of LDL could be observed within minutes of its addition to cells. Unexpectedly, however, transcytosis of LDL continued for at least an additional hour even as the number of intracellular vesicles containing LDL decreased significantly (Fig. 1A). These data suggested that HCAECs were storing LDL at least temporarily after its internalization.

To confirm this, we pulsed HCAECs at 2 sequential time points with LDL tagged with fluorophores of 2 different wavelengths. Cells were first pulsed with DiI(red)-tagged LDL, followed 20 min later by Alexa 488(green)-LDL (Fig. 1B). As before, cells had been depleted of LDLR. We observed transcytosis of both LDL populations within minutes of their addition (Fig. 1C) and significant colocalization of the fluorophores at the base of the cell, suggesting common sites for LDL exocytosis (Fig. 1D). However, we also observed an intracellular pool of DiI-LDL that did not colocalize with Alexa488-LDL (Fig. 1E), particularly away from the basal membrane of the cell. Together, our data indicate that LDLR-*independent* internalization of LDL leads to transcytosis but also the intracellular storage of LDL, a location from which transcytosis continues.

### LDL transcytosis is independent of the classic endocytic maturation pathway

**Fig. 2:**
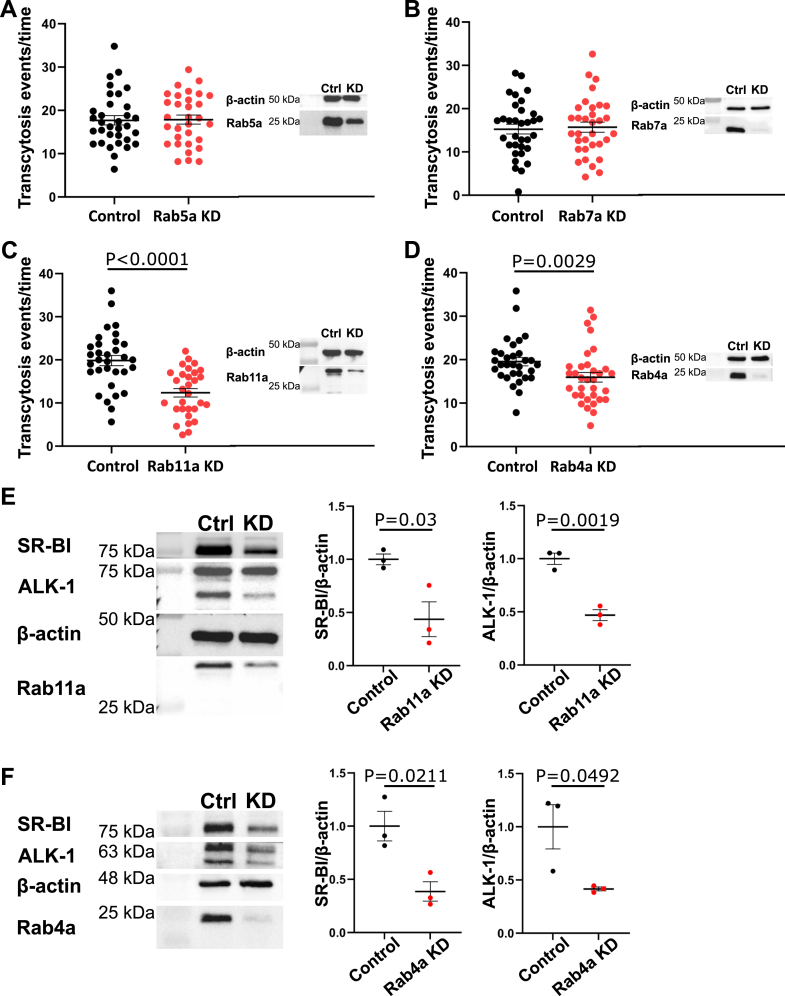
LDL transcytosis does not require Rab5a and Rab7a. A: Depletion of Rab5a had no effect on LDL transcytosis in HCAECs. Quantification of transcytosis events is shown on the left, representative Western blot to confirm Rab5a knockdown is shown on the right. Each point represents one cell. n = 3. B: Depletion of Rab7a did not affect LDL transcytosis. Left side shows quantification of transcytosis events. Right side shows representative Western blot to confirm knockdown of Rab7a. Each point represents one cell. n = 3. C: Depletion of Rab11a decreased LDL transcytosis. Quantification of LDL transcytosis events is on the left, and representative blots are on the right. Each point represents one cell. n = 3. D: Depletion of Rab4a decreased LDL transcytosis in HCAECs. Left shows quantification of LDL transcytosis events, right shows representative blots to confirm depletion of Rab4a. Each point represents one cell. n = 3. E: Western blots were performed to quantify the protein levels of ALK1 and SR-BI after Rab11a knockdown in HCAECs. Representative blots are shown on the left, quantification is on the right. n = 3. F: Western blots were performed to quantify ALK1 and SR-BI protein levels after Rab4a knockdown in HCAECs. Representative blots are shown on the left, quantification is on the right. n = 3. Statistical significance was assessed by Student's *t*-test (A, B, C, E, and F) or Mann-Whitney *U* test (D); error bars represent SEM.

The prolonged residence time of LDL in the endothelial cells raised the possibility that the LDL-bearing compartment might interact with other cellular vesicles or organelles. Classical endocytosis is characterized by the sequential acquisition and replacement on the vesicle of specific Rab GTPases that regulate endosome maturation through the recruitment of effector proteins. For instance, early endosomes acquire Rab5, whose downstream effectors, such as early endosome antigen 1, permit membrane fusion (10). This culminates in the transition from Rab5-containing early endosomes to Rab7-containing late endosomes. To determine if LDL transcytosis intersects with this pathway, we depleted HCAECs of Rab5a by siRNA. Knockdown of Rab5a had no effect on LDL transcytosis (Fig. 2A) despite significantly reducing the uptake of dextran (Supplemental Fig. 1A), consistent with Rab5’s known role in micropinocytosis (11, 12, 13). Similarly, depletion of Rab7a did not alter rates of LDL transcytosis (Fig. 2B), despite inducing an increase in the accumulation of the acidotropic dye LysoTracker (14, 15) (Supplemental Fig. 1B). Thus, LDL transcytosis appears distinct from the canonical route of endosome maturation.

The internalization of LDLR via clathrin-coated pits is notable for the role of recycling endosomes. In this case, progressive acidification of the vesicle leads to dissociation of LDLR from LDL and recycling of the receptor to the plasma membrane. The latter is known to require either Rab4 (fast recycling) or Rab11 (slow recycling) (16). To determine if this route is implicated in LDL transcytosis, we depleted Rab4 and Rab11 separately by siRNA and observed a significant reduction in LDL transcytosis under both conditions (Fig. 2C, D). However, depletion of these Rabs also caused a concomitant reduction in ALK1 and SR-BI protein levels in cell lysates (Fig. 2E, F), suggesting that interference with recycling endosomes had prevented appropriate targeting or recycling of the receptors. Thus, while LDL transcytosis is independent of Rab5a and Rab7a, we can draw no conclusions about the role of recycling endosomes.

### LDL associates with the *trans*-Golgi apparatus during transcytosis

**Fig. 3:**
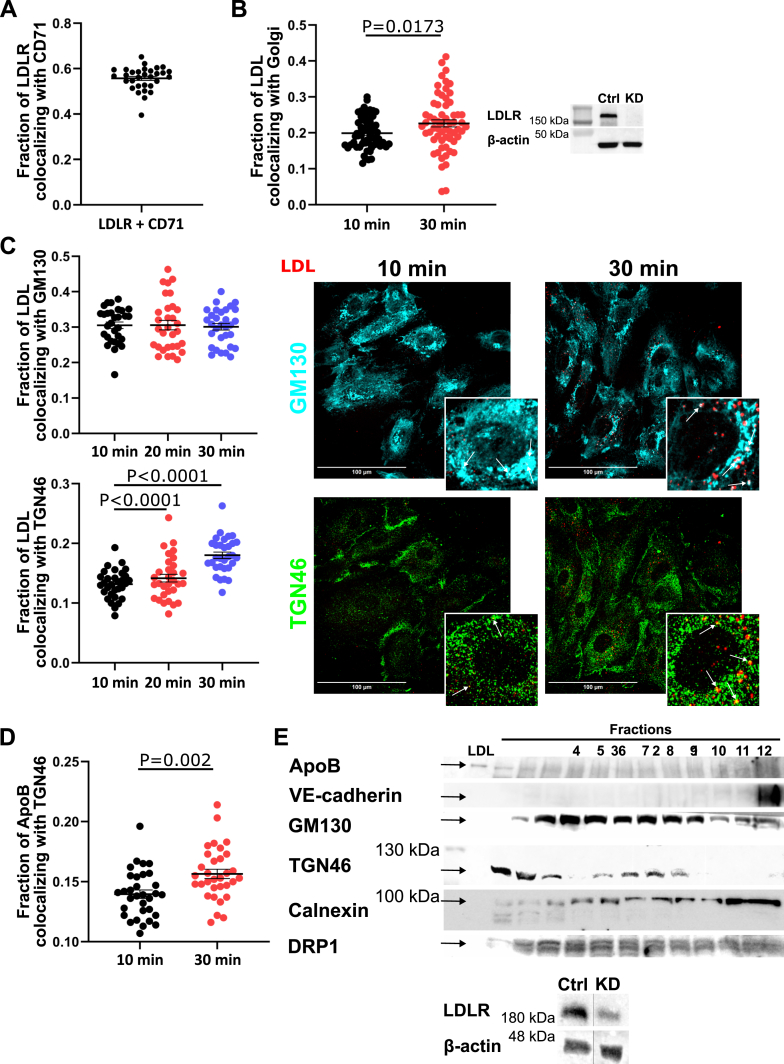
LDL is transported to the *trans*-Golgi network during LDL transcytosis. A: Colocalization between LDLR and CD71 was assessed by immunofluorescence as a positive control (Mander’s coefficient = 0.557). n = 3. B: Colocalization between LDL and Golgi is increased over time in cells depleted of LDLR (Mander’s coefficient for 10 min = 0.199; 30 min = 0.226). Quantification of colocalization is presented on the left. Representative Western blot to confirm knockdown is presented on the right. n = 6. C: Colocalization between LDL and TGN46 increased over time in cells depleted of LDLR (Mander’s coefficient for 10 min = 0.132; 20 min = 0.142; 30 min = 0.180), whereas colocalization with GM130 remained unchanged (Mander’s coefficient for 10 min = 0.305; 20 min = 0.306; 30 min = 0.301). Colocalization was assessed by immunofluorescence microscopy. Quantification of colocalization is on the left, representative images and magnified insets are on the right. Arrows pointing toward areas with colocalization. n = 3. The scale bar represents 100 μm. D: Immunofluorescence was performed to confirm colocalization between ApoB and TGN46 (Mander’s coefficient for 10 min = 0.139; 30 min = 0.156). n = 3. E: LDL was detected in cellular fractions containing the *trans*-Golgi network, but not the endoplasmic reticulum, in HMEC-1 depleted of LDLR. Representative subcellular fractionation western blots are shown on the top and Western blot to confirm knockdown at the bottom; the left-most lane was loaded with LDL as a positive control. n = 2. Statistical significance was assessed by Student's *t*-test (B, D), one-way ANOVA followed by Dunn’s (C; GM130) or Bonferroni’s (C; TGN46) multiple comparison test; error bars represent SEM.

The apparent independence of LDL destined for transcytosis from the endosome maturation pathway led us to consider whether the cellular secretory pathway might be required instead. In other cell types, newly synthesized lipoproteins destined for secretion pass through the Golgi en route to the cell surface (17). To investigate whether LDL traffics through the Golgi during transcytosis, we performed colocalization studies using immunofluorescence microscopy. As a positive control to demonstrate that we could reliably detect and quantify colocalization, we first assessed the overlap between LDLR and the transferrin receptor, CD71, 2 receptors known to cotraffic through the early endocytic recycling pathway (18, 19). As expected, we observed strong colocalization (Fig. 3A). We then measured the degree of colocalization of LDL with the Golgi apparatus in cells depleted of LDLR. We observed partial colocalization of LDL with the Golgi apparatus (Mander's correlation coefficient for LDL to Golgi = 0.199), and this increased significantly over time (Fig. 3B, Supplemental Figs. 2 and 3). Specifically, the colocalization of internalized LDL with the *trans*-Golgi marker TGN46 increased over time while remaining constant with the *cis*-Golgi apparatus marker GM130 (Fig. 3C). To validate this result by another method, we examined the subcellular localization of ApoB-100 by immunofluorescence. As with fluorophore-conjugated LDL, exogenous ApoB-100 colocalized with TGN46, and this increased significantly over time (Fig. 3D).

To determine if the Golgi apparatus is required for LDL transcytosis, we briefly treated HCAEC with BFA, which rapidly disrupts Golgi structure and function (20, 21, 22). Disruption of the Golgi significantly decreased LDL transcytosis, yet had no effect on the internalization of LDL or on the transcytosis of albumin (Supplemental Fig. 4). Thus, the Golgi apparatus is required for the transcytosis of LDL.

Finally, to confirm our immunofluorescence data by a complementary method, we isolated the *trans*-Golgi apparatus biochemically from endothelial cells incubated with LDL and probed lysates for ApoB-100 by immunoblotting; cells had been depleted of LDLR by siRNA prior to the experiment to eliminate confounding from nontranscytotic pathways. One hour after the addition of LDL, we detected ApoB-100 preferentially in cellular fractions containing the *trans*-Golgi but not the endoplasmic reticulum (ER) or the *cis-*Golgi (Fig. 3E). Thus, LDL that was internalized independently of LDLR resides at least in part in the *trans*-Golgi network.

### Transcytosis of LDL requires Rabs 6a and 10

**Fig. 4:**
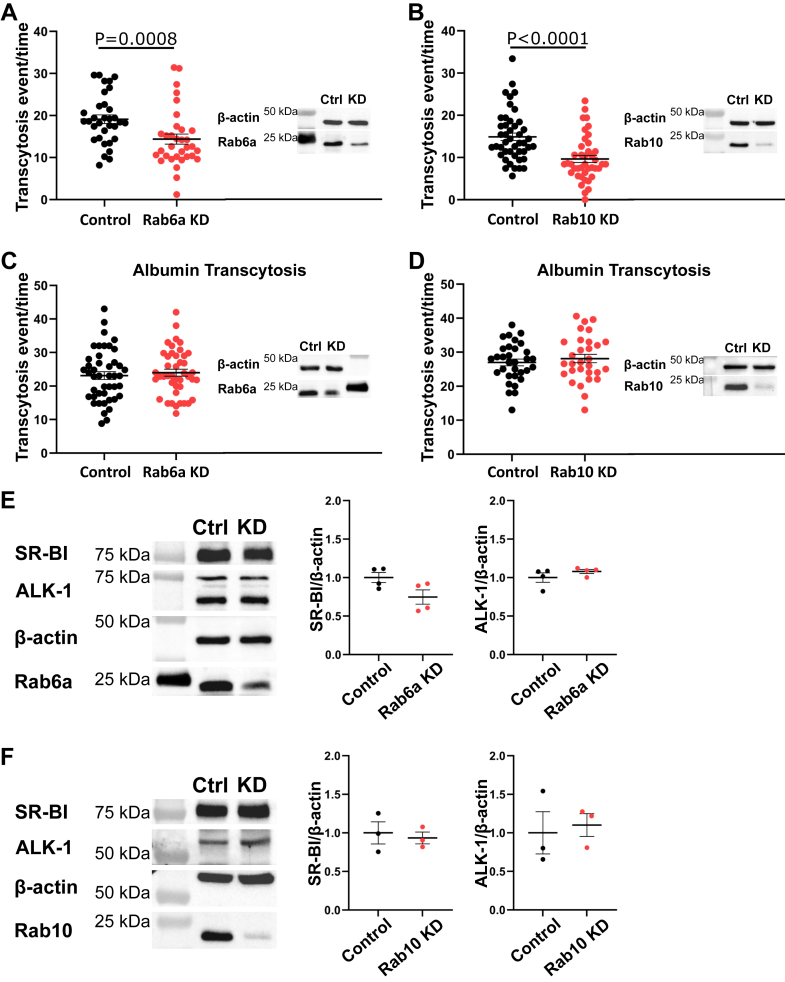
Depletion of Rab6a and Rab10 inhibits LDL transcytosis without reducing SR-BI and ALK1 expression. (A) Depletion of Rab6a or (B) Rab10 in HCAECs decreased LDL transcytosis. Quantification of LDL transcytosis events on the left, and representative blots to confirm knockdown are on the right. Each point represents one cell. n = 3 (Rab6a) or n = 4 (Rab10). C: Transcytosis of albumin remains unchanged upon the depletion of Rab6a or (D) Rab10 in HCAECs. Left shows quantification of albumin transcytosis events, right shows representative blots to confirm Rab6a or Rab10 knockdown. Each point represents one cell. n = 3. E: Western blots were performed to quantify the abundance of SR-BI and ALK1 protein in HCAECS depleted of Rab6a or (F) Rab10. Left shows representative blots, and right shows quantification of the protein levels; n = 4 (Rab6a) and n = 3 (Rab10). Statistical significance was assessed by Mann-Whitney *U* test (A, B) or Student's *t*-test (C-F); error bars represent SEM.

Vesicular traffic to and from the Golgi apparatus is regulated by a distinct set of Rab GTPases, including Rabs 6, 10, and 18. Rab6 is involved in traffic to and from as well as within the Golgi (23, 24, 25), whereas Rab10 has mainly been implicated in exocytosis from the Golgi to the plasma membrane (26, 27). In contrast, Rab18 reportedly directs Golgi traffic retrograde to the ER (28). We found that depletion of Rab6a and 10 by siRNA significantly decreased LDL transcytosis (Fig. 4A, B). By contrast, depletion of Rab18 and Rab8b had no effect (Supplemental Fig. 5).

**Fig. 5:**
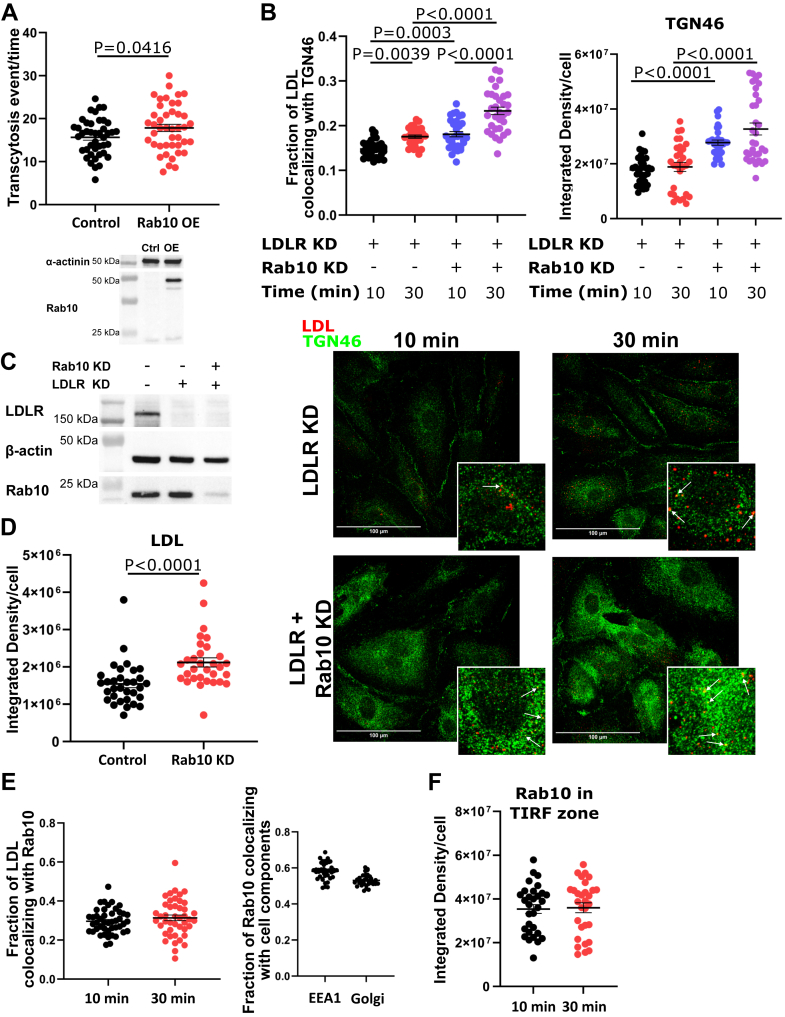
Rab10 regulates LDL exit from the Golgi. A: Overexpression of Rab10 increases LDL transcytosis. Representative Western blot is shown at the bottom to confirm overexpression of Rab10. Each point represents one cell. n = 4. B: HCAECs depleted of LDLR exhibited an increase in colocalization between LDL and *trans*-Golgi network over time, as assessed by immunofluorescence microscopy. Depletion of Rab10 further increased the colocalization between LDL and TGN46 (quantification on the left; Mander’s coefficient for Rab10^+/+^ 10 min = 0.148; Rab10^+/+^ 30 min = 0.175; Rab10^−/−^ 10 min = 0.181; and Rab10^−/−^ 30 min = 0.233) and the intensity of staining for TGN46 (quantification on the right). Representative images and magnified insets at the bottom, arrows pointing toward colocalization between LDL and TGN46. n = 3. The scale bar represents 100 μm. C: Representative Western blot to confirm the knockdown of LDLR and Rab10. n = 3. D: Rab10 depletion does not impair LDL internalization. n = 3. E: LDL does not colocalize with Rab10 over time in HCAECs depleted of LDLR (Mander’s coefficient for 10 min = 0.296; 30 min = 0.314). Colocalization between Rab10 with early endosomes (Mander’s coefficient = 0.581) and Golgi (Mander’s coefficient = 0.531) was independently assessed as positive controls. These coefficients are not mutually exclusive and do not represent proportions of a whole; n = 3 (Golgi) and n = 4 (EEA1). F: Rab10 does not accumulate at the base of the cells depleted of LDLR within the TIRF zone. n = 3. Statistical significance was assessed by Student's *t*-test (A, E, F), one-way ANOVA followed by Bonferroni’s (B; colocalization) or Dunn’s (B; integrated density) multiple comparison test, or Mann-Whitney *U* test (D); error bars represent SEM. EEA1, early endosome antigen 1.

The effect of Rab6 and Rab10 depletion on LDL transcytosis was not because of alterations in either the total level of expression (Fig. 4E, F) or the ability of SR-BI and ALK1 to reach the cell surface (Supplemental Fig. 6). We verified that knockdown of Rab10 or Rab6a had no off-target effects on Rab6a or Rab10 protein levels, respectively (Supplemental Fig. 7).The effect of knockdown was also a ligand-specific effect since transcytosis of albumin was unimpaired (Fig. 4C, D). Finally, overexpression of Rab10 increased LDL transcytosis as well as the number of LDL-containing vesicles at the base of the cell (Fig. 5A, Supplemental Fig. 8).

Next, to determine the function of Rab10 in LDL transcytosis, we quantified the colocalization of LDL with the *trans*-Golgi network after Rab10 knockdown. In cells depleted of Rab10, colocalization of LDL with the *trans*-Golgi increased over time (Fig. 5B, C), suggesting that Rab10 may be required for the exit of LDL from the Golgi. Accordingly, depletion of Rab10 did not impair the internalization of LDL (Fig. 5D). Furthermore, we observed a significant increase in the size of the *trans*-Golgi network in cells depleted of Rab10 (Fig. 5B), as measured by immunofluorescence.

Finally, we wondered if the vesicles bearing LDL were also characterized by an association with Rab10 during their passage through the cell. However, colocalization of LDL with Rab10 remained modest and unchanged over time (Fig. 5E). Rab10 also did not accumulate at the base of the cell as assessed by TIRF microscopy (Fig. 5F). Together, this suggests that while Rab10 is involved in the exit of LDL from the Golgi, it is not transported directly with LDL-bearing vesicles.

## Discussion

The intracellular route taken by LDL during transcytosis is largely uncharacterized. The bulk of existing literature has focused on the requirement for specific receptors at the apical endothelial surface. Both SR-BI and ALK1 have been demonstrated to mediate LDL transcytosis and to contribute to atherogenesis (2, 3); in contrast, the high-affinity LDLR is not typically required (2, 4). Beyond this, the fate of LDL after its internalization for transcytosis is obscure. By comparison, the intracellular events following internalization of the LDLR are well understood: LDL is contained in Rab5-positive early endosomes, which progressively acidify during maturation. This acidification leads to the dissociation of the receptor from LDL, recycling of LDLR to the cell surface, and progression of LDL into Rab7-containing late endosomes (29, 30). However, the involvement of SR-BI and ALK1 instead of LDLR in the initial stages of LDL transcytosis suggests the possibility that the intracellular route taken during transcytosis may be distinct.

Our data indicate that LDL transcytosis proceeds rapidly upon internalization and continues for at least an hour afterward. The detection of persistent transcytosis was not because of recycling of exocytosed LDL, as the media were changed at each time point; in addition, the number of LDL-containing vesicles contained in the cell (as measured by TIRF) decreased steadily over time, even as the rate of transcytosis remained relatively constant. Furthermore, when LDL tagged with distinct fluorophores was added to the cell at 2 different time points, transcytosis of both types of LDL could be detected, but their colocalization in the cell was incomplete. Together, these data suggest for the first time that upon internalization, LDL transcytosis may proceed directly as well as indirectly, the latter from an intracellular niche. Our data implicate the *trans*-Golgi network as a critical component of this niche.

Our pulse-chase experiments were performed in cells depleted of LDLR in order to eliminate confounding from any nontranscytotic LDLR-mediated internalization. This raises the concern that alteration of endocytic traffic by depletion of LDLR might have confounded our results. However, acute inhibition of the Golgi apparatus using BFA in cells retaining LDLR significantly reduced LDL transcytosis; in contrast, the transcytosis of albumin was unchanged. The internalization of LDL was also unaffected by BFA. These data rule out confounding by cellular toxicity and support a role for the Golgi in LDL transcytosis even in the presence of LDLR.

As vesicular traffic is regulated by a large family of Rab GTPases, we reasoned that only a specific set of Rab GTPases was likely required for LDL transcytosis. We found that LDL transcytosis is independent of the classic endosome maturation pathway, as depletion of Rabs5a and 7a had no effect. Consistent with this notion, we have previously reported that inhibition of lysosomes does not impair LDL transcytosis (31). Although knockdown of Rab4 and Rab11 reduced LDL transcytosis, it also led to decreased protein levels of SR-BI and ALK1. Given that Rab4 (32, 33) and Rab11 (34, 35) are well-established regulators of receptor recycling and trafficking to the plasma membrane, we speculate that their loss disrupts the membrane targeting of SR-BI and ALK1. This prevents us from drawing any definitive conclusions about the specific role of recycling endosomes in LDL transcytosis. Recent studies have shown that components of the COMMANDER pathway, including COMMD proteins and the WASH complex, are required for the recycling of both LDLR and SR-BI (36, 37). Further investigation is needed to determine whether this pathway may contribute to receptor-mediated LDL transcytosis.

The involvement of the Golgi in LDL transcytosis suggested which Rabs to examine next. While both Rab6a and Rab10 have been implicated in Golgi traffic, their functions overlap only partially. Rab6a regulates all stages of pre-, intra-, and post-Golgi traffic (23, 24, 25), whereas Rab10 has been reported to regulate traffic from the Golgi to the plasmalemma (26, 27). Consistent with this, depletion of Rab10 increased the accumulation of LDL in the *trans*-Golgi network and led to an increase in the size of the Golgi. This suggests that Rab10 is regulating the exit of LDL from the *trans*-Golgi, perhaps analogous to its role in regulating the targeting of other post-Golgi cargo like TLR4 to the plasma membrane (38). As others have shown that the depletion of Rab10 does not directly alter Golgi morphology (39, 40, 41), we interpret the accumulation of LDL in the *trans-*Golgi of Rab10-depleted cells as a consequence of a ligand-specific trafficking defect rather than generalized Golgi dysfunction. Consistent with this model, we found that depletion of the *cis*-Golgi regulator Rab18 (28) had no effect on LDL transcytosis. Further work is ongoing to elaborate on the role of Rab6a, as well as other Golgi-associated Rab proteins (e.g., Rab9), in regulating LDL transcytosis.

To our knowledge, this is the first report of the storage of LDL in the endothelial *trans*-Golgi network, an organelle known to be enriched in caveolin-1 (42). The purpose of Golgi targeting is unclear, and we cannot exclude another intermediate or penultimate destination en route to exocytosis at the base of the cell. Another caveat is that the sensitivity of our approach (i.e., immunofluorescence) is limited, and interactions of LDL with other organelles like the ER cannot be excluded. We believe that interactions of LDL with the *trans*-Golgi and other organelles may permit exchanges of cholesterol and fatty acids, and this is currently under investigation.

Our experiments using LDL tagged with distinct fluorophores revealed extensive colocalization of the LDL at the basal membrane, even when the fluorescent species were added 20 min apart. This observation suggests the possibility of common exocytosis sites at the basal membrane of the endothelial cell. If true, the mechanism is unclear and will require further study.

In conclusion, we have defined an intracellular pathway taken by LDL that is independent of LDLR and regulates transcytosis. The transcytotic pathway proceeds both directly and indirectly and intersects with the *trans*-Golgi network, where at least a fraction of LDL appears to be stored; this process is regulated by Rabs 6a and 10. Exit from the Golgi toward the endothelial basal membrane requires Rab10. Our findings suggest the possibility that transcytotic LDL-containing vesicles interact with the cell during transit and may thereby influence cellular metabolism. Given recent data highlighting its importance to the development of atherosclerosis (4, 43), understanding its regulation (44) has the potential to uncover novel therapeutic targets.

## Data availability

All data are available in the article or supplemental data.

## Supplemental data

This article contains supplemental data.

## Conflict of interest

The authors declare that they have no conflicts of interest with the contents of this article.
