# Supplementary figures and images for: LDL transcytosis passes through the trans-Golgi network and requires Rab10
Source: J Lipid Res. 2025 Sep 2;66(10):100893. doi: 10.1016/j.jlr.2025.100893 (PMC12510203; doi:10.1016/j.jlr.2025.100893)

**A**

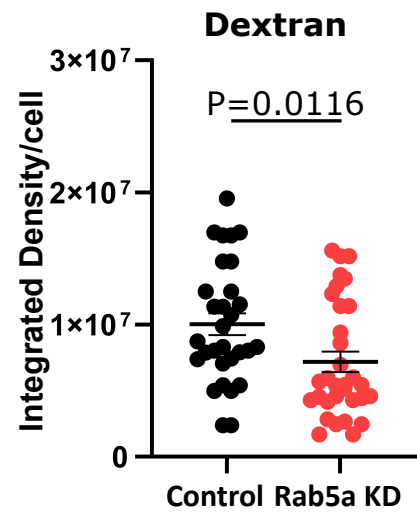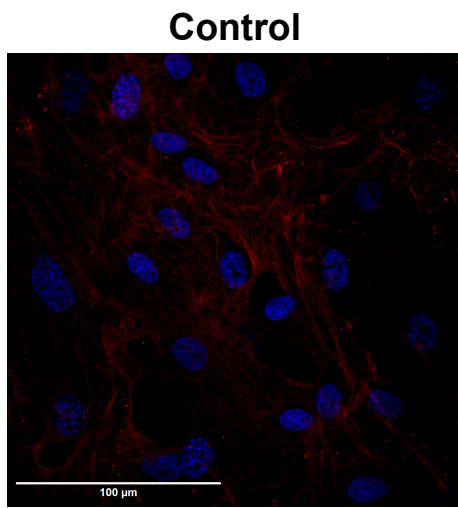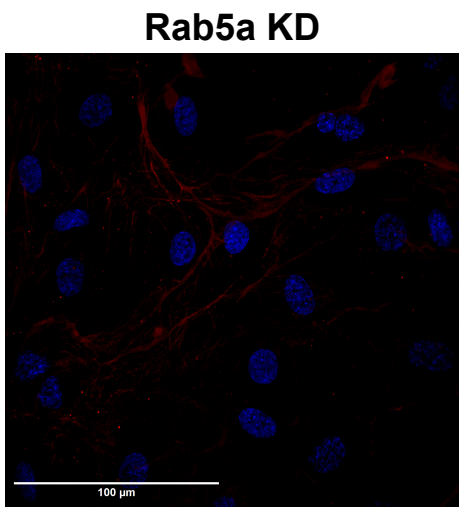

**B**

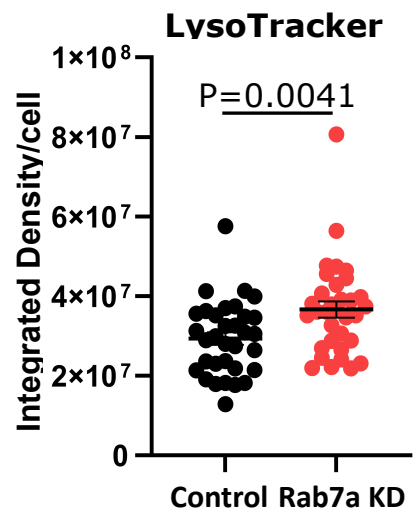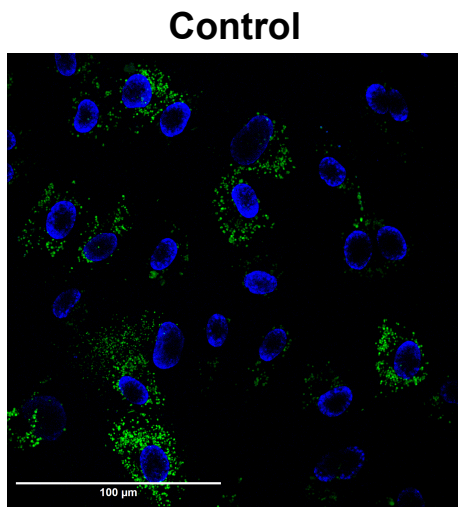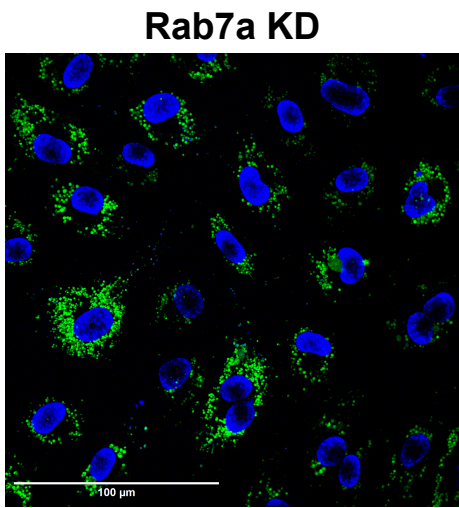

Supplement: Supplemental Fig. 1 [file mmc1.pdf]

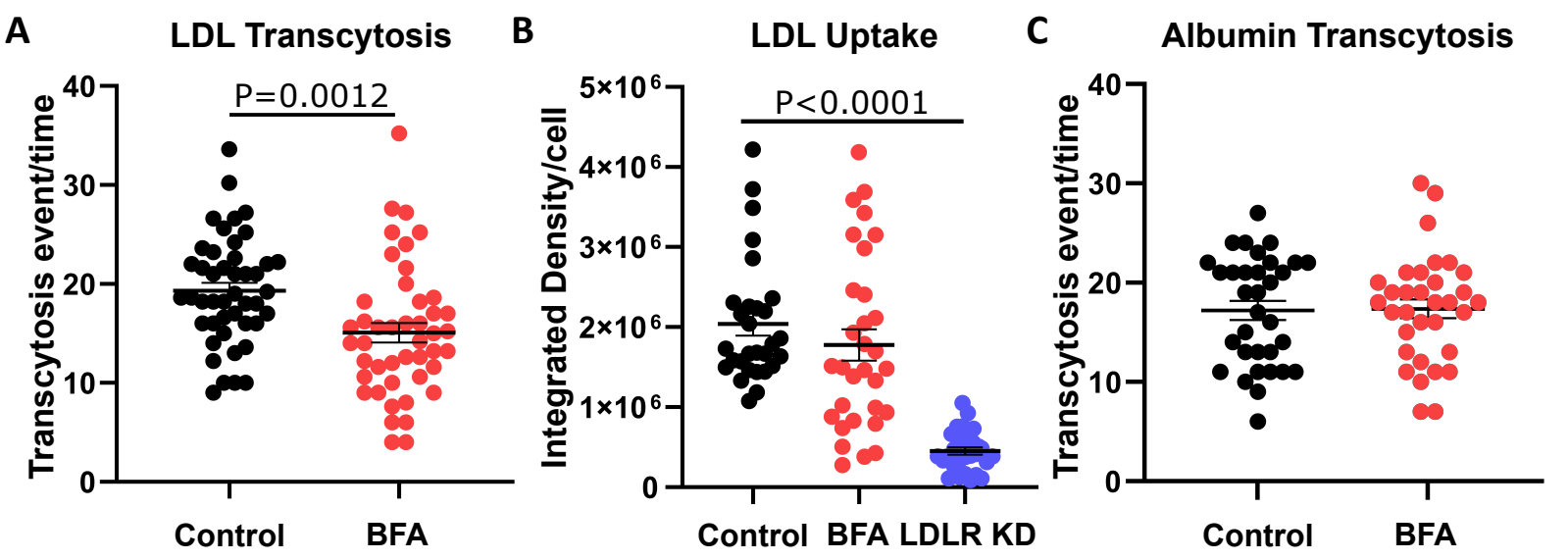

Supplement: Supplemental Fig. 4 [file mmc4.pdf]

**A**

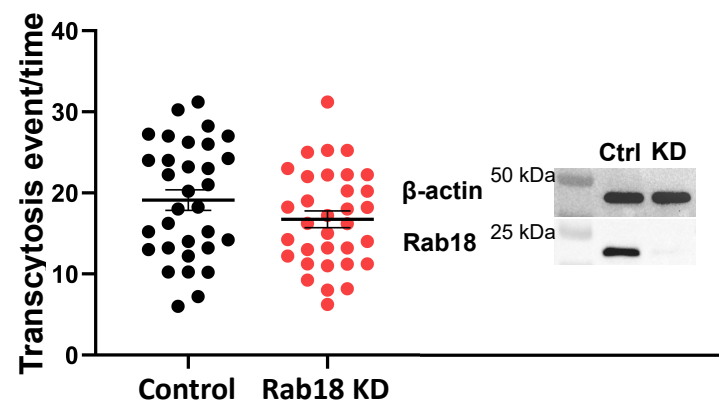

**B**

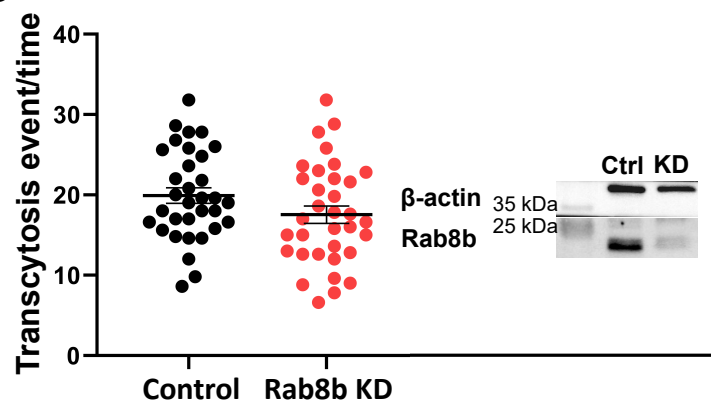

Supplement: Supplemental Fig. 5 [file mmc5.pdf]

**A**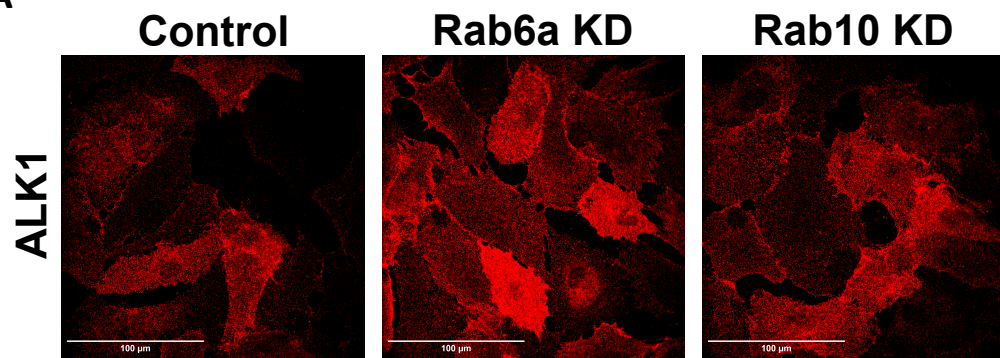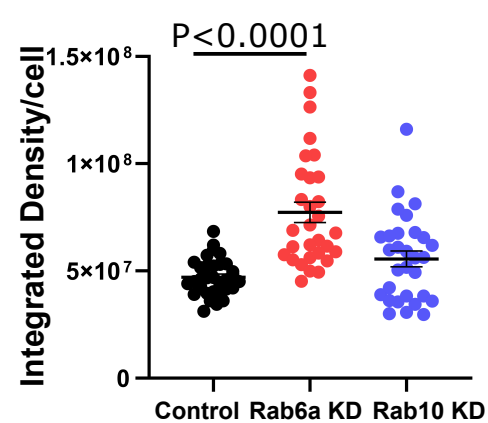**B**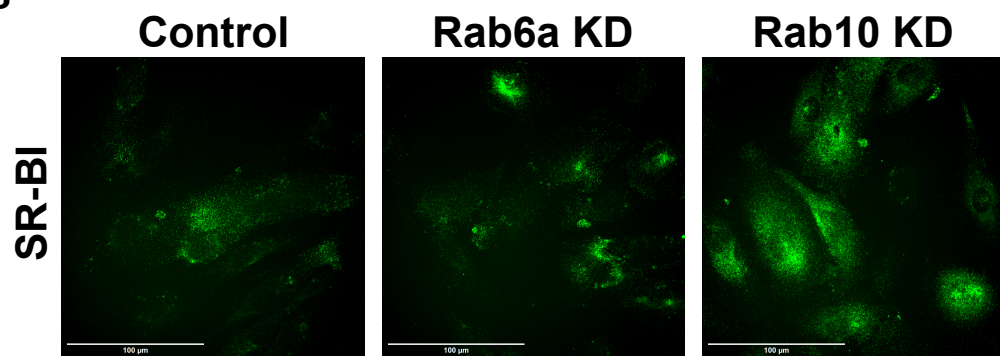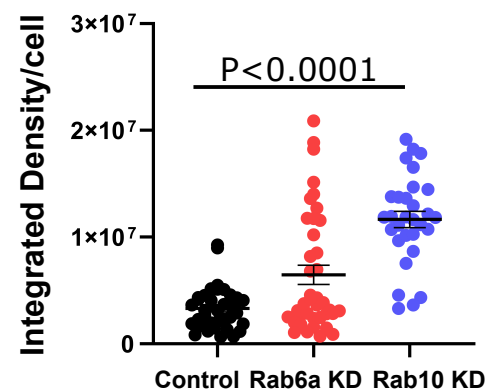

Supplement: Supplemental Fig. 6 [file mmc6.pdf]

**A**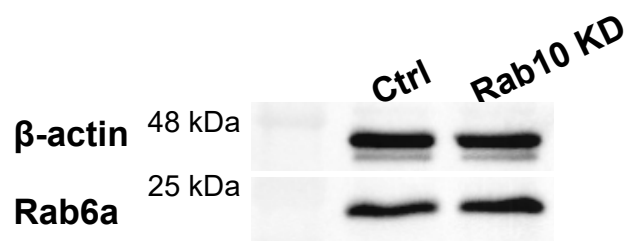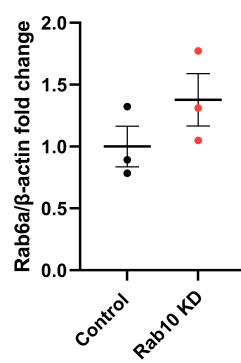**B**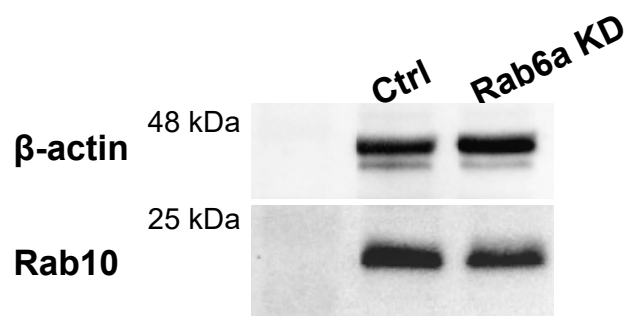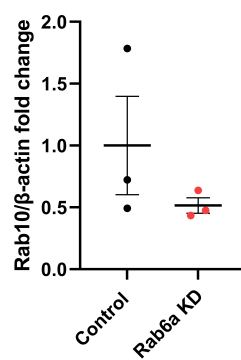

Supplement: Supplemental Fig. 7 [file mmc7.pdf]

**A**

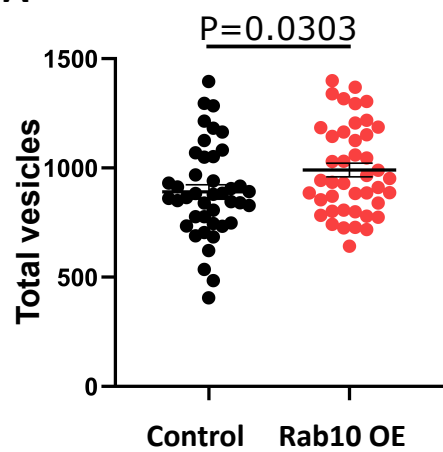

Supplement: Supplemental Fig. 8 [file mmc8.pdf]
