# Supplementary material for: LDL transcytosis passes through the trans-Golgi network and requires Rab10
Source: J Lipid Res. 2025 Sep 2;66(10):100893. doi: 10.1016/j.jlr.2025.100893 (PMC12510203; doi:10.1016/j.jlr.2025.100893)
Supplement: Supplemental Figure Legends [file mmc9.docx]

Supplemental Figure 1. Functional validation of Rab5a and Rab7a knockdown in HCAECs

**A,** Depletion of Rab5a reduced dextran uptake, confirming impaired early endocytic activity. Each point represents one cell. Quantification is on the left, representative images are on the right. n=3, scale bar is 100 µm. **B,** Rab7a knockdown increased LysoTracker signal, consistent with disrupted late endosomal–lysosomal maturation. Each point represents one cell. Quantification is on the left, representative images are on the right. n=3, scale bar is 100 µm. Statistical significance was assessed by Mann-Whitney U-Test; error bars represent SEM.

Supplemental Video 1. Three-dimensional rendering of LDL and TGN46 staining at 10 minutes in HCAECs depleted of LDLR. LDL is in red, TGN46 is in green.

Supplemental Video 2. Three-dimensional rendering of LDL and TGN46 staining at 30 minutes in HCAECs depleted of LDLR. LDL is in red, TGN46 is in green. Note the increased association of LDL and the trans-Golgi over time compared to Supplemental Figure 2.

Supplemental Figure 4. Disruption of the Golgi decreases LDL transcytosis

**A,** Disruption of the Golgi with Brefeldin A (BFA) decreases LDL transcytosis. Each point represents one cell. n=4. **B,** BFA inhibition had no effect on LDL uptake or **C,** albumin transcytosis. n=3. Statistical significance was assessed by Student t-test (A, C) or 1-way ANOVA followed by Dunn’s multiple comparison test (B); error bars represent SEM.

Supplemental Figure 5. LDL transcytosis does not require Rab18 or Rab8b

**A,** Depletion of Rab18 in HCAECs had no effect on LDL transcytosis. Left shows quantification of transcytosis events, right shows representative western blot to confirm Rab18 knockdown. Each point represents one cell. n=3. **B,** Depletion of Rab8b had no effect on LDL transcytosis. Quantification of transcytosis events on the left, representative western blot to confirm knockdown on the right. Each point represents one cell. n=3. Statistical significance was assessed by Student t-test; error bars represent SEM.

Supplemental Figure 6. Depletion of Rab6a or Rab10 does not interfere with ALK1 or SR-BI’s ability to reach the cell surface

**A,** Cell surface immunofluorescence staining (i.e. without membrane permeabilization) was performed to confirm that Rab6a or Rab10 depletion do not interfere with ALK1’s ability to reach the cell surface. Left shows representative images, right shows quantification of ALK1’s staining intensity. n=3, scale bar is 100 µm. **B,** Cell surface immunofluorescence staining of SR-BI upon Rab6a or Rab10 depletion. Representative images are on the left and quantification is on the right. n=3, scale bar is 100 µm. Statistical significance was assessed by 1-way ANOVA followed by Dunn’s multiple comparison test; error bars represent SEM.

Supplemental Figure 7. Depletion of Rab6a or Rab10 does not affect protein expression of Rab10 or Rab6a, respectively.

**A,** Western blot to quantify protein expression of Rab6a upon the depletion of Rab10 in HCAECs. Representative blot on the left, quantification of western blot result on the right. n=3. **B,** Western blot to quantify protein expression of Rab10 upon the depletion of Rab6a in HCAECs. Left shows representative blot, right shows quantification. Statistical significance was assessed by Student t-test; error bars represent SEM.

Supplemental Figure 8. Overexpression of Rab10 increases LDL trafficking to the base of the cell

**A,** Overexpression of Rab10 increases the number of LDL-containing vesicles at the base of the cell (TIRF zone). Each point represents one cell. n=4. Statistical significance was assessed by Student t-test; error bars represent SEM.
